# Normothermic regional perfusion in paediatric donation after circulatory determination of death—the Oxford position statement from ELPAT

**DOI:** 10.3389/frtra.2024.1320783

**Published:** 2024-01-29

**Authors:** J. Brierley, A. Pérez-Blanco, J. Stojanovic, N. Kessaris, A. Scales, A. Paessler, N. Jansen, A. Briki, D. Gardiner, D. Shaw

**Affiliations:** ^1^ Paediatric Intensive Care and Bioethics Centre, Great Ormond Street Hospital for Children NHS Foundation Trust, London, United Kingdom; ^2^ National Transplant Organization, Madrid, Spain; ^3^ Department of Paediatric Nephrology and Transplantation, Great Ormond Street Hospital for Children NHS Foundation Trust, London, United Kingdom; ^4^ Department of Nephrology and Transplantation, Guy's Hospital, Guy's and St Thomas' NHS Foundation Trust, London, United Kingdom; ^5^ NHS Blood and Transplant, Bristol, United Kingdom; ^6^ Policy Department, Dutch Transplant Foundation, Leiden, Netherlands; ^7^ Intensive Care Unit, Nottingham University Hospitals NHS Trust, Nottingham, United Kingdom; ^8^ Department of Health, Ethics & Society, Maastricht University, Maastricht, Netherlands

**Keywords:** donation after circulatory determination of death (DCD), normothermic regional perfusion (NRP), paediatric, ethics, law, psychosocial

## Introduction

Following the recent report of the use of both abdominal and thoracoabdominal normothermic regional perfusion in paediatric donation after circulatory death (DCD) donors in Spain (1, 2), the area needs urgent ethical deliberation and clarification. For those involved in paediatric donation and transplantation the public acceptability of any novel approaches needs careful consideration, given the implications of negative publicity, especially in countries where healthcare innovations can be portrayed in the media in misleading ways (3).

This report results from a joint working group session of the Paediatric Donation and Transplant and Deceased Donation groups of Ethical, Legal and Psychosocial Aspects of Organ Transplantation (ELPAT), a section of the European Society of Organ Transplantation (ESOT).

## Normothermic regional perfusion (NRP)

Normothermic regional perfusion is a relatively new post-mortem intervention used in the donation after circulatory determination of death (DCD) donor. In NRP, after circulatory determination of death an extracorporeal membrane oxygenation (ECMO) circuit is used to restores oxygenated circulation of blood inside the donor (also known as in-situ machine perfusion), effectively restoring function to perfused organs. NRP isolated to the abdominal organs only, via an ascending aortic clamp or intravascular balloon inflation, is known as A-NRP. When NRP restores the circulation to both thoracic and abdominal organs, via operative clamps, surgical ties or leaving vessels open to the atmosphere, it is known as TA-NRP. Both techniques are designed to isolate the brain from the restored circulation, but in TA-NRP, the heart will recommence beating inside the donor (4).

There has been ongoing international debate about NRP in adult donors (5, 6). The benefits are both reduced warm ischaemic damage to organs and that the intervention permits a post-mortem period of stable in-situ perfusion, allowing surgeons to assess organ viability over time rather than in a non-functional and hypoxic state. NRP is supported by a growing evidence base, which suggests that by minimising the overall warm ischaemic damage providing more and higher quality organs, the best evidence being for liver transplantation (7, 8).

However, ethical concerns have been expressed about NRP, specifically the risk of invalidating the very definition of death, and therefore the dead donor rule (9). For example, in countries that adhere closely to the USA Uniform Determination of Death Act (UDDA), where death is defined as the “irreversible cessation of circulatory and respiratory functions”, restoring circulation after death could be said to invalidate this definition. Of interest, NRP use is increasing in the USA despite the wording of the UDDA. In Australia, the legal definition of death concerns the irreversible cessation of circulation in the body; therefore, NRP is currently not used as it appears to be unlawful (10).

Many national ethics bodies are debating the permissibility of NRP, as highlighted in the USA (6, 7), though variability in the definition of death may mean NRP is permitted in some countries but not others. An international collaborative group is arguing for a unified brain-based concept of death, which, if accepted would means that it is only the circulation to the brain that is relevant for defining death (9, 11). In this context, if the cerebral circulation is isolated, then the definition of death holds despite any restoration of non-cerebral circulation. However, concerns have been expressed about the risk of cerebral perfusion despite occlusion of the main cerebral arteries, via the spinal arteries and posterior cerebral circulation (6). An additional concern, more prominent in North America than Europe, is that the act of vessel clamping, or ligation becomes the cause of death (5, 6).

The joint working group session heard that this concern has led to a moratorium or pause on TA-NRP in both the Netherlands and the UK, though A-NRP continues in adults as the risk of restoring cerebral circulation is considered far lower (10). In contrast, in Spain both modes of NRP have been performed successfully in both child and adult DCD donors, with rigorous audits showing adherence to the national protocols. The Spanish national DCD heart transplant protocol (includes children and adults) mandates clamping and venting the three aortic arch arteries to the atmosphere before TA-NRP starts, and donor-monitoring with transcranial Doppler or evoked potentials or another *ad hoc* technique to measure blood brain flow or brain function from withdrawal of life-sustaining treatment to the end of the NRP (12).

**Figure 1:**
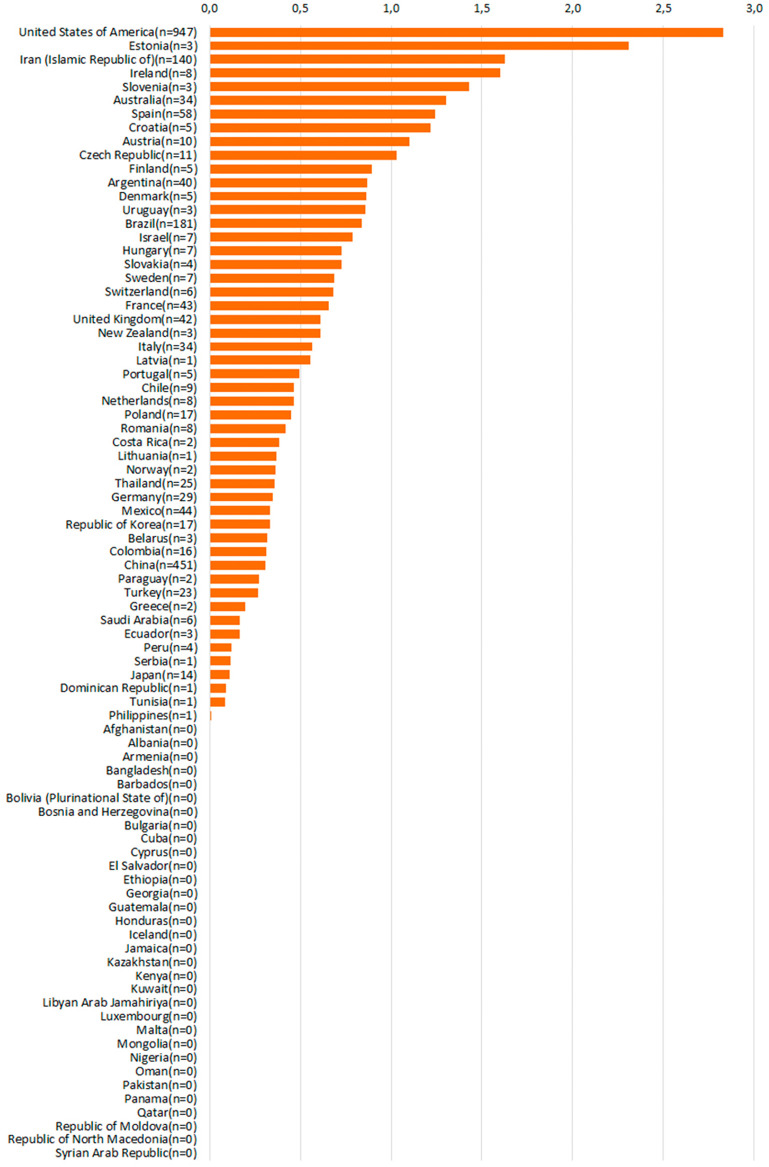
Absolute numbers of actual pediatric donors, 2022. Source: Global Observatory on Organ Donation and Transplantation, data provided upon direct request, www.transplant-observatory.org.

**Figure 2:**
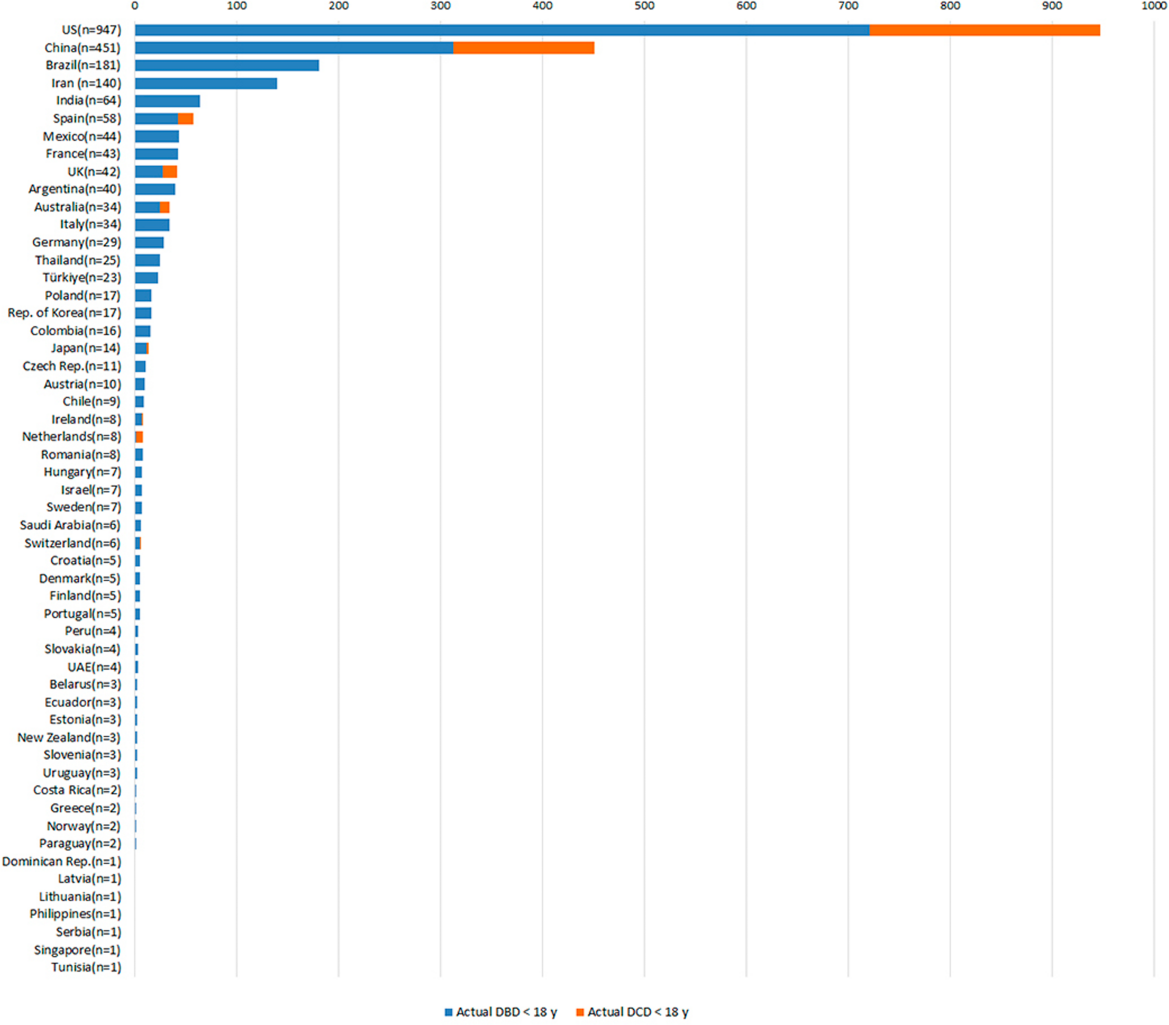
Absolute numbers of actual pediatric donors (total, pDBD and pDCD), 2022. Source: Global Observatory on Organ Donation and Transplantation, data provided upon direct request, www.transplant-observatory.org/.

The need for organs for child-recipients is clear, and sadness at the great number of children dying when a transplant could have saved them is only worsened by data on countries different performances in paediatric organ donation (Figures 1, 2). The reasons for the loss of potential paediatric organ donors is multifactorial, and occurs at every step of the donation process (13).

So, what are the specific potential benefits of *paediatric* NRP? There are very limited published studies analysing the influence of P-NRP in the utilization and recovery of organs of paediatric DCD donors (pDCD). Moreover, the outcome of recipients and grafts transplanted from pDCD donors with NRP is restricted mainly to single-centre retrospective series and individual experiences.

Transplant teams show reluctance to accept organs obtained from pDCD, resulting in higher discard rates compared to grafts recovered from paediatric DBD donors. The main reasons suggested for this are damage to donated organs due to the warm ischaemic time that follows the withdrawal of life supporting therapies (WLST) and post-transplant complications, e.g., ischaemic cholangiopathy, vascular thrombosis, and acute kidney injury (14–18).

Using *in situ* P-NRP with extracorporeal membrane oxygenation (ECMO) soon after the declaration of death has been shown to increase the recovery as well as the utilization of grafts from pDCD compared with the rapid recovery technique (RR). Benefits are related to intra-operative assessment of the graft and NRP capacity to regenerate the ischaemic tissue, diminishing post-transplant complications (19). Miñambres et al. (1), achieved 85% of utilized p-DCD liver grafts obtained with P-NRP (without ex-situ perfusion) and 100% of recovered kidney grafts from p- DCD compared to 0.5% of utilized p-DCD liver grafts obtained with RR (20).

In adult recipients, NRP with ECMO is associated with superior liver and kidney graft short-term function compared with grafts of DCD donors recovered without NRP (21).

Incorporating thoracic P-NRP in heart transplantation has shown successful outcomes at 1 year follow up in ABO non-compatible and compatible, broadening transplantation opportunities for small children and reducing wait list mortality (2, 22).

A recently published multi-visceral transplantation with P-NRP and ECMO marked a milestone in the history of abdominal transplantation. High susceptibility of the intestine to ischaemia had led transplant teams to refrain from performing transplants from p-DCD donors, resulting in prolonged waiting list time for patients, leading to developmental delays and high mortality. A total of 3 multi-visceral transplants with P-NRP have been performed in the same centre with successful outcomes in 2022–23 (23).

What does the success of NRP in pDCD child donors in Spain (1, 2), the first paediatric NRP reported, mean for other countries? In children, given the variability in the DCD-donor from term infant of a few kilograms to adult-sized teenagers, and the greater cerebral blood flow relative to cardiac output in smaller children (24), it might be argued that greater technical expertise is required to reliably isolate the cerebral circulation, especially in infants. Conversely, the Spanish experience is of easier clamping and control due to the proximity of the vessels and the need for less dissection to isolate them. However, across Europe, would all retrieval teams have the expertise to deliver safe and reliable isolation of the cerebral circulation in the various sizes of pDCD donors in every referral hospital without the investment in strict protocols and donor monitoring the Spanish have instigated?

## DCD in Europe

The acceptability of DCD *per se* varies in Europe, with some countries not permitting it at all, with even greater restrictions on its use in children (25), Given this cultural and international variability, it is unsurprising that even where DCD is practised, the ethical acceptability of NRP varies.

As a European society, we consider an ethical viewpoint from the ELPAT group necessary, and we hope helpful, and can find no previous ethical or legal publications about NRP in children.

## Discussion

The Paediatric Donation and Transplant and Deceased Donation groups of ELPAT held a workshop to discuss NRP in children in Oxford in 2023. Initially, the groups reviewed different national approaches to NRP, and the concerns expressed. There was reflection on dying being a process with the concept of death being at the point of permanence, with acknowledgement of the historical term “irreversible”. The point of permanence was clarified as the time at which the dying process will not be reversed, typically following a prior decision to withdraw life-sustaining treatment with the child being not for resuscitation. The group then discussed recent international attempts to unify the cerebral and circulatory definitions of death around the loss of brain function (9, 26).

There is a physiological relationship between cerebral flow, perfusion and function, where loss of brain function is the definitional endpoint goal. However, the group acknowledged the lack of a currently accepted method to reliably establish loss of brain perfusion or function after circulatory (cardio-respiratory) arrest. Instead, the surrogate “loss of circulation” is employed as cerebral function ceases rapidly (within 30 s) after circulatory arrest (27). Clearly, therefore, restoring cerebral circulation in a DCD donor risks invalidating the entire determination of death.

TA-NRP entails occluding cerebral blood supply from the thoracic aorta, specifically the brachiocephalic arteries, to isolate the cerebral circulation from the perfusion commenced via an aortic cannula (28). Anatomically, the concerns involve the risk of cerebral perfusion by various arterial routes not isolated by the clamping.

Although there is no evidence of this occurring in either animal models or humans (29, 30), the group did not consider the existing paediatric data as robust as might be expected before the introduction of a new technique in something as crucial as deceased donation.

Ex-situ machine perfusion, an alternative to NRP for DCD-heart retrieval, was discussed. It provides an alternative in countries where TA-NRP is currently prohibited, offering a successful mode of transplant in the medium term in both adults (31) and children (32). However, the use of available devices, such as the OCS system for donor hearts, is currently restricted to donors >50 kg and recipients >30 kg (33), and furthermore the licenced devices are expensive. The use of novel non-proprietary solutions without age or weight restrictions is being explored, but at present for paediatric heart transplant recipients under 30 kg, a large proportion, outside Spain there is little alternative to a DBD organ.

Many countries do not list children with comorbidities, such as chromosomal anomalies or multiple chronic organ dysfunction, as the chance of, for example, a heart transplant is so remote. This is the situation irrespective of any consideration about the long-term outcome of their comorbidities. The mortality rates of children on the waiting list are not insignificant, due to the burdens of invasive organ support such as ECMO or VAD, which include infection and neurological injury associated with prolonged anticoagulation.

The groups then directly heard about the Spanish NRP experience, which has transformed deceased donor organ availability. Whilst multifactorial, a critical element has been retrieval teams' willingness to attend now that organs can be assessed whilst perfused at body temperature i.e., in a functioning state in what once was considered marginal situations. Due to this and other innovations, the liver transplant waiting lists for children and adults in Spain have almost been cleared. This achievement was lauded, but concerns were expressed in the group about the broader European acceptability of TA-NRP programmes in children when, in several countries, there is a moratorium or pause in adults due to scientific and ethical concerns.

Specific discussion surrounded public acceptability and lack of conclusive data regarding the ability to isolate the cerebral circulation in the donor (both adult and paediatric). The group also discussed the acceptability of transcranial dopplers, Bispectral Index (BIS), EEG and other neurophysiological techniques not validated for this purpose in children and the inability to evaluate the brainstem which is most at risk from low flow collateral circulation.

In something as important as determining human death, professional and public confidence is vital. For many, but certainly not all in the group, this meant that a precautionary principle approach should be adopted regarding any interventions to facilitate donation that may, even theoretically, influence the dying process and reliable death determination. Especially in countries influenced by previous media misreporting, broad professional and public acceptability, and safety information are required before new processes can be introduced (3). Adopting this principle, there seems inadequate data currently about the risk of cerebral perfusion in adults, specifically in TA-NRP, to support NRP unreservedly and certainly little in children. Evidence in animal models is emerging, albeit after TA-NRP commenced in humans (34). Given the concerns about the technical and organizational challenges involved, our consensus was that more data and broader debate is required before we could recommend extending NRP to other countries that practice paediatric DCD. However, data will hopefully soon emerge from the Spanish experience, where monitoring of the cerebral circulation data from child donors should soon be available.

We acknowledge that other concerns have been raised, some by those from groups opposed many organ donation norms, such as the “permissibility of DCD” and the “acceptability of neurological determination of death”. Their arguments include that clinicians are inducing brain death in a living person by preventing cerebral blood flow when the circulation restarts in TA-NRP, and also that restarting the circulation in the donor, irrespective of isolating the cerebral vessels, invalidates the dead donor rule (35, 36). Such concerns are not limited to those groups expressing faith or world-view-based concerns, with concerns also raised in mainstream scientific journals (37).

However, it is crucial to recognise the tragic on-going mortality in children on the transplant waiting list and of those not even listed due to the lack of suitable organs. When seen in this context given the Spanish experience, determining data in deceased child donors to inform ethical discourse, ought to be prioritised, particularly those furthest away from adult size, i.e., infants and small children.

Whilst anticipating data about immature animal NRP models and Spanish paediatric data, there is need for broad consensus regarding innovations in deceased organ donation. The groups agreed that national professional consensus among those working in donation and transplantation should be established, based on robust science and organ retrieval governance structures, before the public is presented with a fair question in countries that consider introducing paediatric NRP, especially TA-NRP.

The final point involved possible expansion of ex-situ organ perfusion, following the impressive UK paediatric DCD heart program which similarly could change the transplantation paradigm. Indeed, if developed to its potential it could one day allow donated organs to be treated and repaired for days, in essence the first step to growing organs outside the human body. Such efforts will need considerable investment in research and medical engineering. Even the current systems are relatively expensive, at around 65,000 Euros, which will preclude their use in many countries. Furthermore, the OCS system may be limited to countries where DCD organ removal follows a five-minute post-arrest stand-off time, so for countries with a longer time, such as Italy at twenty-minutes, its utility is unclear.

## Summary

Where paediatric DCD is practised, but not NRP, reliable data on the ability of the various NRP approaches to isolate the cerebrall circulation should be a priority, especially thoracoabdominal NRP given the lack of thoracic organs is such an acute issue. In the interim or where NRP remains unacceptable, ex-situ perfusion could be expanded, especially if cheaper systems without donor weight restrictions can be developed.

Our proposal is that suitable juvenile animal data and the emerging Spanish experience, should be used to inform ethical deliberation, public debate and professional consensus. However, given children are dying waiting for a transplant, this should be given national priority.
